# On the Action of Medicines

**Published:** 1848-03

**Authors:** 


### ECLECTIC DEPARTMENT,

### AND SPIRIT OF THE MEDICAL PERIODICAL PRESS.

---

*On the Action of Medicines.* By James Blake, M. D., Professor of Anatomy in the St. Louis University.

In the memoir, published in the last number of this Journal,* I brought forward a number of experiments which prove that as regards all the most rapidly fatal poisons, a sufficient interval always elapses between their application to any part of the body, and the appearance of the first symptoms of their action, for them to be carried to the brain through the medium of the circulation, and therefore that the theory of the action of poisons by means of impressions made on the extremities of the nerves, is opposed to the facts furnished us by these observations; for, did poisons act as is generally believed, through the medium of impressions made on the nerves, they ought to produce some symptoms of their action immediately on their application; and the interval, should any imaginary interval be required for the transmission of a nervous impression, could not be much longer in one animal than in another —whereas, the facts, brought forward in my last paper, show that

* See January No. of Buffalo Medical Journal.

there is a difference of twelve seconds between the time that elapses between the application of a poison and the appearance of the first symptoms of its action, according as the experiment is made, on a horse or on a rabbit. It is perfectly opposed to all our knowledge of the functions of the nerves, to suppose that any such difference can exist between different animals in the time required for an impression to be propagated along a nerve; in fact we have every reason to believe, that impressions received at one extremity of a nerve are as instantaneously perceived at the other, as is the electrial communication established through hundreds of miles by means of the electric telegraph—and that it involves almost an absurdity to suppose that a nervous impression should be sixteen seconds traveling from a horse's neck to his brain.

I shall now, however, enter on the consideration of another class of facts, which have been considered by some as affording positive evidence that poisons act only when they are brought into contact with those organs, the functions of which they affect—whilst by others, the deductions that most obviously arise from them, have been attempted to be set aside, by certain objections on account of the manner in which the experiments have been performed. It is not necessary that I should enter into many particulars on this part of the subject, as the experiments of Magendie, on the absorption of poisons, are probably known to all your readers. I shall merely relate two experiments, from amongst many that I have performed on this point, because I think they furnish the most striking evidence that could be brought forward, that a poison cannot produce any effect withont its being carried to the nervous centres, even though it should be absorbed. From the facility with which the stomach can be isolated from the other parts of the body, as regards the blood passing over it, without its nervous connexions with the system being at the same cut off, I have, in these experiments, made use of this organ as the point on which to apply the poison. The abdomen of a dog was opened and a ligature passed round the æsophagus at its cardiac termination. The vena porta was also included in another ligature, so that when this was tightened no blood circulated over the stomach, which was thus completely cut off from the rest of the system, as regards at least, the circulation, although many of its nervous communications remained unimpaired. The ligature around the vena porta was tightened, and three drachms of hydrocyanic acid were introduced into the stomach, through an opening made in its parieties. The animal remained apparent totally unaffected by the poison for the space of ten minutes, although it is evident that a large dose of a most powerful poison was thus brought into contact with a great extent of surface, liberally supplied with nerves, and those nerves the nerves of organic life, on which poison are supposed more particularly to act: after the poison had been in the stomach without producing any symptoms for ten minutes, the ligature around the vena porta was loosened, for a short time, and the animal immediately began to show symptoms of the action of the poison: the ligature around the vein was again tightened, so as to prevent the blood containing the poison, from circulating over the system. The quantity of poison, however, that had already entered the circulation, had been sufficient to arrest respiration, and the animal would undoubtedly have died had not artificial respiration been resorted to: after this had been continued for about ten minutes, the effects of the poison had so far gone off, that respiration was again performed naturally, showing the interesting fact, that an animal could be recovered from the effects of poisoning with hydrocyanic acid, with large doses of the poison still in the stomach, the poison being prevented from mixing with the blood circulating through the system. This experiment is certainly the most striking that I have performed on the absorption of poisons, for not only do we find that an animal remained unaffected by a large dose of hydrocyanic acid in the stomach, as long as it was prevented entering into the blood and being carried over the system, but we even see it recovering from the effects of a dose of poison, sufficient to have destroyed life, whilst there was still nearly three drachms of hydrocyanic acid in the stomach.

Objections, however, have been brought forward to the evidence furnished by the last experiment, conclusive as it must appear, on the grounds, that, whilst the blood was prevented circulating over the parieties of the stomach, its nerves were not in a state to propagate any impression that might be made on them. In order to remove this source of fallacy, should it exist, the following experiment was devised, in which it will be seen, that the nerves of the stomach whilst in contact with the poison, were in their natural condition as regards the circulation of the blood: a tube was inserted into the hepatic artery of a dog, the point of the tube looking towards the part where the artery comes off from the cœliac axis, so that any fluid injected through the tube would pass backwards into the cœliac axis, and thus become mixed with the blood circulating over the stomach and intestines. A ligature was passed round the vena porta, but was not tightened; the circulation over the surface of the stomach and intestines was thus carried on in its natural state, with the exception of the slight abstraction afforded by the ligature of the hepatic artery. A solution, containing seven grains of nitrate of strychnia, was injected, through the tube, in the hepatic artery, and thus became mixed with the blood circulating over the viscera; at the moment the injection was introduced the ligature round the vena porta was tightened, and an incision made into the vein on the visceral side of the ligature, so as to allow the blood, that was circulating over the viscera, to escape: by this means the blood containing the poison was allowed to circulate freely over the stomach, whilst, at the same time, it was prevented from entering into the general circulation. Under these circumstances no symptoms of the action of the poison manifested themselves for some minutes, and certainly not at the time that the blood containing it, was circulating over the vicera, and whilst it was in contact with the nerves witn which they are so freely supplied. The animal died in about five minutes, from the effects of poison and from loss of blood; but I have no doubt but that the poison had been absorbed, from the blood which had escaped from the vena porta, and which ran into the cavity of the abdomen. It is quite evident that the poison did not produce any impression on the nerves of the viscera, capable of giving rise to symptoms of poisoning, although it was in a sufficient quantity to have killed the animal in a few seconds. The facts brought forward in these last experiments, are, I think, sufficient to prove that a poison might be brought into contact with a large surface of the body, abundantly supplied with nerves, and yet that no symptoms of its action will manifest themselves, provided it be prevented from becoming directly applied to the nervous centres, by blood circulating through them.

We have only now to notice some experiments that have been brought forward, in a work on the action of Poisons, published a few years since, by Addison and Morgan, and which have been frequently appealed to, in proof that some poisons can produce general effects from an impression made on the nerves of the part to which they are directly applied. The most important experiment adduced by these gentlemen, in support of the local action of a poison, is that in which the jugular vein of a dog was exposed in a considerable part of its course: two ligatures were applied to it, three inches apart; a portion of woorara was introduced into that part of the vessel comprised between the ligatures, and the upper ligature was then removed. The blood was thus brought in contact with the poison which became applied to the parietes of the vein. In these circumstances, it is stated, that the poison rapidly produced its effects: and it is concluded that these effects were owing to an impression made on the nerves of the isolated portion of the vein. I would observe, however, that there must evidently be some fallacy in this experiment; for had the vein been pefectly isolated in the space comprised between the two ligatures, the only parts by which it still remained connected with the system were at those parts of the vessel beyond the ligatures, and thus as far as nervous communication was concerned, it was in the same condition as if it had been entirely removed from the body, for a ligature still was around the lower part of the vessel, and one had previously been placed around the upper portion, an operation quite sufficient to incapacitate the nerves, which had been included in it, from performing their functions, and thus there remained no means by which an impression, made on the nerves, in the isolated portion of the vessel, could be transmitted to the nervous centres. As this obvious source of fallacy must have struck the authors of the experiment, did it really exist, I conclude that the vein could only have been partially separated from the surrounding tissues. In this case we have a ready explanation of the action of the poison: the free anastomoses which exist between the veins of opposite sides readily permitting the poison to beceme mixed with the blood circulating through the body. Even supposing the complete isolation of the vein, the ready manner in which solutions become diffused through fluids, would forbid the conclusion that the solution of the poison in the blood could be confined to the isolated portion of the vessel, it must speedily become mixed with the blood in the upper part of the vessel, above the point at which it was isolated, and thus could readily enter the general circulation.

Another experiment brought forward by these gentlemen to prove that a poison does not exert its effects by being taken into the blood, and thus applied to the nervous centres, is one in which they connect the carotid arteries of two dogs, in such a manner, that they suppose the blood from the heart of the one dog is sent to the brain of the other, and then, having inserted poison into the abdomen of the animal, the inferior ends of whose carotids are connected with the superior extremities of the carotids of the other they conclude that if the poison is absorbed into the blood, the second animal should feel its effects as well as the first, on account of the blood, containing the poison, passing into its carotids from the carotids of the dog to which the poison has been applied. This supposition, however, is entirely opposed to the physical arrangement of the vessels that supply the brain: were the carotids the only vessels going to this organ, the experiment would be a fair one; but, in the dog, the vertebral arteries are so large as readily to furnish a sufficient supply of blood to the brain, even when the carotids are tied. The only condition in which the blood could pass from one animal to the other is, by the pressure of the blood on the parietes of the superior end of the carotid of each dog being less than the pressure on the inferior portion of the carotid of the dog with which it was connected, and the quantity of blood that passed would be in proportion to the difference of pressure on the two ends of the tubes connecting the vessels. I have ascertained, by direct experiment with the hemadynamometer, that the pressure exerted on the parietes of a tube, inserted into the distant extremity of the carotid, is less by a column of mercury of not more than two or three tenths of an inch, than the pressure on a tube inserted into the end nearer the heart. If we suppose, therefore, that the pressure in the arterial system of each animal, was originally equal, (a fact by no means probable,) it is evident that but a very small quantity of blood can pass from the arteries of one animal to those of the other, and this only whilst both are in the same state. As soon as the poison begins to exert its influence on either animal, the pressure in its arterial system will be diminished, and thus, far from blood containing the poison being sent to the brain of the sound animal, the only effect of this arrangement will be to cause a reflux of pure blood, from the arteries of the sound dog, into those of the poisoned one. It is not surprising, that under these circumstances, the experiment furnished a negative result, It is evident, that from neither of these experiment, can any argument be deduced in favor of the local action of poisons.

Having now disposed of the evidence which has been brought forward in support of the local action of poisons, and from analogy for the local action, also, of a very large class of substances, used as medicinal agents, I shall offer a few remarks, on one or two practical points, on which the above facts have a direct bearing. It has been a question, which has often been discussed, and which, in many instances, has been decided against the evidence furnished by the above experiments, as to whether a person, who was known to have swallowed a very large dose of any of the more rapidly fatal poisons, could, after taking it, be able to perform certain acts indicating the possession of consciousness and voluntary motion. If, for instance, a person who had swallowed an ounce of strong hydrocyanic acid, might be able to cork the bottle from which it had been taken, to walk any distance, to place themselves on a bed, or, in fact, perform any other act indicating the possession of consciousness and the power of voluntary motion. Now, if we are to believe in the instantaneous action of these powerful poisons, we must answer this question in the negative; and, in those cases in which it can be proved that a person has swallowed a large dose of poison, and that, subsequently to taking it, we find the bottle corked, for example, or we find a person on a bed, at some distance from the spot where the vessel containing the poison had been placed, we are led to consider that some other party must have been present, and thus suspicion would naturally be thrown on any person who might have been present. A review of the facts brought forward in these memoirs will prove how erroneous would be any evidence based on such considerations—for even with large doses of the most rapidly fatal poisons. an interval of from twelve to fifteen seconds would elapse before any effect could be produced and, therefore, that if the individual, who had swallowed the poison retained sufficient presence of mind, there would be ample time for acts indicating consciousness and the perfect possession of the powers of voluntary motion to be performed; the interval, undoubtedly, is short, but I find, that at the ordinary rate of walking, it would be sufficient for a person to walk eighteen or twenty yards, or to perform any act not requiring more time than twelve to fifteen secouds to be accomplished in. To show how erroneous are the opinions, even of medical men, on this point, it is but a few years since, that in a trial before the jury in England, four medical men out of five, gave it as their opinion, that the fact of the bottle being found corked, and the deceased lying on a bed, in a case in which three drachms of hydrocyanic acid had been swallowed by a girl, necessarily proved that some person must have been with the deceased at the time the poison was taken; fortunately, however, in this instance, the medical evidence was disregarded, and the accused individual escaped; but it is probable, that cases have happened, in which more weight has been attached to the opinions of those whose duty it is to be conversant with facts of this sort.

The bearing of the facts here brought forward, on our therapeutical and physiological theories, would tend to render us more circumspect, in attributing the effects, of a large class of remedies, to any local action they may exert on the nerves of the part to which they are applied, and a knowledge of the fact that substances are generally absorbed into the blood, before they exert any marked action on the system, will direct our inquiries into the changes they give rise to in this fluid, or to the tissues of the different organs to which they become applied. It is always an advantage, in the solution of any problem, to define, by a careful analysis, the limits or the exact nature of the question to be solved, and, in no instance, is this more necessary than in elucidating the complicated phenomena presented to us by physiology and pathology. The question as to whether it is by the changes that these poisons effect in the blood, or whether merely by an action that they exert on the central points of the nervous system, or whether, in fact, it is by changes which take place, both in the blood and in the nervous centres, that these substances produce their effects, is a point which I have not yet been able to determine, and I fear it must be left undecided until the attention of physiologists shall have been, in some degree, diverted from the mere material or physical structure of the organism, to an investigation of the more important chemical properties of the solids and fluids. Simple observation is not sufficient to enable us to discover the properties of these elements; as well might the chemist attempt to discover the properties of oxygen, by simply observing the phenomena of ordinary combustion, as for the physiologist to expect to arrive at a knowledge of the properties of the various elements of which the living organism is composed, by simply observing the natural processes in which these processes are called into play. It is only by following the experimental method, a method that has furnished such brilliant results in the study of inorganic chemistry, that we can hope to throw any light into the dark arcana of the living organism : from this source, and this source alone, I am convinced we are destined to receive the only light that can guide us in our researches into the complicated properties going on in living bodies, and it is this branch of physiology that I would recommend to the consideration of those of your readers, whose object it is advance that science, which can be the only sure basis of a rational system of therapeutics.—*St. Louis Med. and Surg. Jour.*

